# Embolisation d’un faux anévrisme artériel sur rein unique: à propos d’une complication rare de la néphrolithotomie percutanée

**DOI:** 10.11604/pamj.2020.35.60.14626

**Published:** 2020-02-28

**Authors:** Traore Abdoulaye Ababacar, Alaoui Lamrani Youssef, Alami Badreeddine, Boubbou Meryem, Maaroufi Maaroufi, Kamaoui Imane

**Affiliations:** 1 Service de Radiologie du Centre Hospitalier Universitaire Hassan II Fès, Université Sidi Mohammed Ben Abdellah, Faculté de Médecine et de Pharmacie de Fès, Fès, Maroc; 2 Service de Radiologie du Centre Hospitalier Universitaire Mohammed VI Oujda, Oujda, Maroc

**Keywords:** Percutaneous nephrolithotomy, one kidney, computed tomography, false aneurysm, embolization, Néphrolithotomie percutanée, rein unique, tomodensitométrie, faux anévrisme, embolisation

## Abstract

La néphrolithotomie percutanée s’accompagne d’un risque de complications en particulier hémorragiques, qui en fait une technique potentiellement invasive. Nous rapportons le cas d’une patiente de 70 ans, traitée auparavant pour néphrolithotomie percutanée d’une lithiase sur un rein unique gauche. Une hématurie persistante de moyenne abondance est apparue il y a 2 mois et demi, et qui a motivé à la réalisation d’une tomodensitométrie découvrant un faux anévrisme intra rénal polaire inférieur, d’origine iatrogène. Il a nécessité une embolisation sélective efficace à la colle biologique.

## Abstract (en)

Percutaneous nephrolithotomy is a potentially invasive technique associated with a risk of complications, in particular bleeding. We report the case of a 70-year old female patient who had only left kidney and with a history of renal calculi treated with percutaneous nephrolithotomy. Persistent, average abundant haematuria had occurred two and a half months before, requiring computerized tomography (CT) scan. This had showed false iatrogenic intrarenal inferior polar aneurysm. It required effective selective embolization of biological glue.

## Introduction

La néphrolithotomie percutanée (NLPC) représente l’alternative thérapeutique la plus importante dans la prise en charge des calculs de grande taille ou complexes du rein, ne relevant pas de la lithotripsie extra corporelle ou de l’urétéroscopie souple [1, 2]. La NLPC sur rein unique est reconnue d’une grande sécurité et les résultats sont comparables avec ceux obtenus sur des reins fonctionnels. Sa réalisation nécessite de grande précaution pour éviter les complications de néphrectomies [3–6]. En effet, la NLPC sur un rein unique constitue un challenge pour l’urologue, pour préserver la fonction rénale et éviter une lésion vasculaire qui peut engager le pronostic vital. L’étiologie des complications hémorragiques tardives de la NLPC sur un rein unique est peu documentée dans la littérature. Nous rapportons dans les suites d’une NLPC, un cas rare d’un pseudo anévrisme artériel sur un rein unique fonctionnel embolisé avec succès.

## Patient et observation

Patiente âgée de 70ans, suivi pour un rein unique gauche fonctionnel, ayant bénéficié d’une NLPC, il ya 45 jours, pour une lithiase rénale gauche. Les suites opératoires étaient simples. Elle est adressée actuellement dans notre structure pour prise en charge d’une insuffisance rénale aigue associée à une hématurie macroscopique d’abondance minime et d’une issue de collection par le trajet pariétal de néphrotomie réalisée. A l’examen clinique, la patiente est apyrétique, sans signe d’hématurie visualisé, cependant sur le trajet du geste opératoire ont observe une fistule pariétale lombaire. Le bilan biologique réalisé, montre un taux d’hémoglobine bas à 9.9g/dl, les leucocytes à 18,5µ/L, la créatininemie à 17mg/l, l’urée à 0,78g/l et le temps de prothrombine diminué à 75%. L’échographie rénale d’urgence a montré une dilatation modérée des voies excrétrices sans obstacle lithiasique ni retentissement morphologique rénale. Ainsi, une sonde urétérale double J est montée pour le drainage urinaire, sanctionné d’une bonne évolution biologique de la fonction rénale.

**Figure 1:**
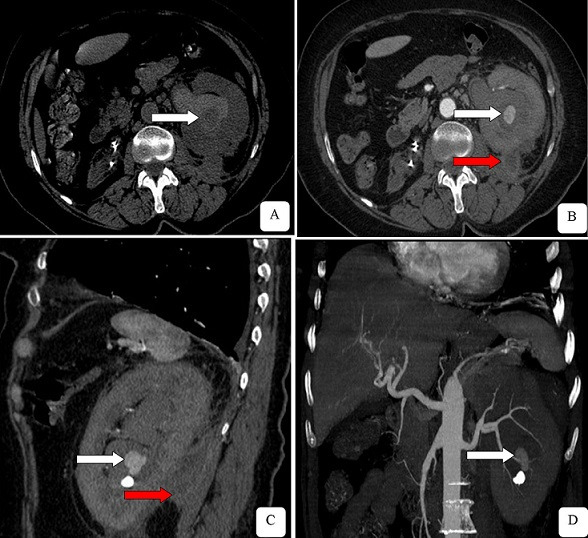
Coupes TDM axiales en contraste spontané (A), et après injection de contraste aux temps artériels (B), en reconstructions sagittale (C) et coronale (D), objective un anévrisme de l’artère inter lobulaire gauche, mesurant 10mm de grand axe (flèche blanche) associé à une fistule néphro-pariétale (flèche rouge); à noter une lithiase calicielle rénale, non obstructive

**Figure 2:**
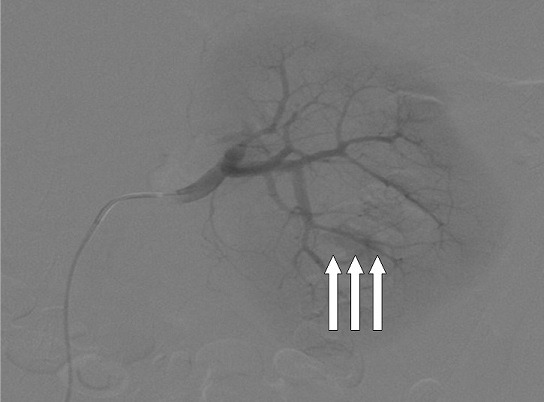
Image d’artériographie post embolisation à la colle biologique montrant l’exclusion efficace du faux anévrisme intra rénal polaire inférieur

Après un mois, l’évolution clinique est marquée par l’apparition et l’aggravation de l’hématurie de moyenne abondance s’accompagnant d’un retentissement hémodynamique clinique et biologique, une déglobulisation revenue à 8,8g/dl ainsi que l’aggravation de la fonction rénale avec une créatininémie à 52mg/l. L’altération du tableau a nécessité la réalisation d’une tomodensitométrie (TDM) abdominale avec injection de produit de contraste iodé, bien tolérée. Elle objective un anévrisme d’origine iatrogène de l’artère inter lobulaire faisant 10mm de grand axe associé à un hématome sous scapulaire et une fistule néphro-pariétale lombaire. Il s’y associe également une collection péri rénale et une lithiase calicielle inferieure non obstructive (Figure 1). La patiente a bénéficié d’urgence d’une embolisation sélective à la colle biologique du faux anévrisme intra rénal polaire inferieur (Figure 2). Le geste thérapeutique est sanctionné d’un tarissement progressif de l’hématurie et une amélioration progressive du bilan biologique. Dans les suites d’une évolution favorable, la patiente est déclarée sortante avec réhydratation orale et un suivi en consultation externe. Une nouvelle TDM de contrôle à 3 mois ne révèle pas de signe de complication ou de récidive.

## Discussion

La néphrolithotomie percutanée est une technique moins invasive que la chirurgie ouverte pour traiter les lithiases rénales, elle n’est pas dépourvue de morbidité [1, 2]. Les complications de la NLPC sont évaluées à 26% en moyenne, incluant des complications mineures et des complications majeures qui peuvent être hémorragiques, urinaires, liées à des lésions des organes de voisinage, infectieuses ou métaboliques [3–7]. Le rein unique représente un modèle particulier d’étude de la fonction rénale après une NLPC. En effet, il existe une dégradation de la fonction rénale chez près de 40% des patients ayant un calcul coralliforme avant tout acte thérapeutique sur un rein unique [8]. Les complications de la NLPC représentent une préoccupation majeure chez les patients présentant un rein unique par rapport à ceux ayant les reins normaux [3–6].

Selon les séries, le taux global de complications de la NLPC sur rein unique est estimé entre 16-27% [9]. Ces résultats sont semblables à une étude à grande échelle réalisée chez les patients avec des reins habituels [10]. En effet, la NLPC est une approche utile pour des calculs rénaux de plus de 20mm de diamètre, notamment chez les patients aux reins uniques [3, 11]. Ce qui vraisemblablement avait conduit à la procédure initiale de prise en charge de notre patiente. Cependant, cette technique est associée à des risques plus élevés de complications graves par rapport à d’autres traitements [12]. Les complications les plus redoutables de la NLPC sont d’ordres hémorragiques; elles restent rares de l’ordre de 2,3%. L’hémorragie peut survenir à plusieurs temps de l’acte, en per-opératoire, en post-opératoire immédiat et à distance. Il s’agit de fistules artério-veineuses, des faux-anévrysmes ou des plaies artériolaires. En occurrence, l’apparition à distance de la NLPC d’une hématurie nécessite le plus souvent un geste d’hémostase, allant jusqu’à la néphrectomie [7]. L’hématurie macroscopique a été le symptôme annonciateur de découverte du faux anévrisme de notre patiente. Dans une étude rétrospective portant sur la NLPC, les auteurs ont rapporté comme principal facteur de risque d’hémorragie massive post-NLPC, la ponction du calice supérieur, le calcul coralliforme, les ponctions multiples, le manque d’expérience du chirurgien et la présence d’un rein unique [13].

Le bilan d’imagerie est nécessaire, si l’hématurie microscopique est associée à l’un de ces signes de choc, clinique ou mécanisme évocateur ou s’il existe une hématurie macroscopique [7]. Actuellement, la TDM sans et avec injection de produit de contraste iodé peut donner des renseignements précis sur la topographie, la densité et la taille des calculs rénaux, et permettre aussi une évaluation pré-opératoire des rapports vasculaires avec l’axe du calice contenant les calculs ou permettant d’y accéder [1, 2]. Au-delà, elle fera le diagnostic des complications vasculaires et de recherche une plaie des voies urinaire. L’anévrisme décrit une image d’addition, de rehaussement intense superposable aux structures artérielles [4, 5]. Dans notre observation, la TDM a attesté rapidement le diagnostic du faux anévrisme intra rénal et du bilan locorégional. Par ailleurs, l’échographie doppler permet également l’étude morphologique rénale et du pédicule vasculaire. Elle peut détecter l’anévrisme rénal sous forme d’une formation liquidienne siège d’un flux doppler artériel. Elle est inutile en période post-thérapeutique récente, car l’air intra-cavitaire rend très difficile le repérage lésionnel [14].

L’émergence des techniques de radiologie interventionnelle a changé la prise en charge de ses complications hémorragiques sévères de la NLPC [15]. L’artériographie qui est une technique invasive permet au radiologue de réaliser simultanément le diagnostic étiologique de l’hémorragie et le traitement d’urgence par une embolisation sélective à la colle ou avec un coïl [16]. L’exclusion du faux anévrisme, à une colle biologique a été l’attitude thérapeutique efficace dans notre observation, aboutissant à une amélioration de la fonction rénale.

Néanmoins, une rechute est possible par revascularisation ou par récanalisation des anomalies vasculaires (10%) pouvant justifier alors une nouvelle embolisation [16]. L’embolisation sélective peut cependant avoir un retentissement sur la fonction rénale après une NLPC [15]. Notre patiente s’est améliorée favorablement sur le plan clinique et biologique avec un recul d’une année. Le risque de séquelles à long terme de l’embolisation hypersélective est donc faible et reste sans comparaison avec le préjudice de la néphrectomie d’hémostase qui était le risque majeur avant l’utilisation de l’embolisation [16].

## Conclusion

La découverte d’un faux anévrisme artériel sur un rein unique est une complication tardive, exceptionnelle dans les suites d’une néphrolithotomie percutanée. La tomodensitométrie est la technique d’imagerie rapide de choix pour le diagnostic positif et de complications. Dans le but de préserver la fonction de l’appareil urinaire, l’embolisation sélective occupe une place majeure dans la prise en charge intrinsèque des faux anévrismes d’un rein unique.

## Conflits d’intérêts

Les auteurs ne déclarent aucun conflit d’intérêts.
